# Prevalence of disorders of gut–brain‐interaction in pediatric patients with in‐remission inflammatory bowel disease: An Italian multicenter study

**DOI:** 10.1002/jpn3.70386

**Published:** 2026-02-23

**Authors:** Giovanna Quatrale, Niccolo’ Chirico, Luigi Colecchia, Giuseppe Stella, Francesco Proli, Pietro Pisano, Erminia Romeo, Paola De Angelis, Licia Pensabene, Giovanni Marasco, Giovanni Di Nardo, Giovanni Barbara, Antonio Gasbarrini, Franco Scaldaferri, Valentina Giorgio

**Affiliations:** ^1^ Department of Neurosciences, Mental Health and Sensory Organs (NESMOS) Sapienza University of Rome Rome Italy; ^2^ Department of Woman and Child Health and Public Health Fondazione Policlinico Universitario “A. Gemelli” IRCCS, Catholic University of Sacred Heart Rome Italy; ^3^ IRCCS Azienda Ospedaliero Universitaria di Bologna Bologna Italy; ^4^ Department of Medical and Surgical Sciences University of Bologna Bologna Italy; ^5^ Gastroenterology and Nutrition Unit, Bambino Gesù Children's Hospital, IRCCS Rome Italy; ^6^ Department of Medical and Surgical Sciences, Pediatric Unit Magna Graecia University Catanzaro Italy; ^7^ Gastroenterology and Endoscopy Unit, Department of Pediatric Specialties Santobono Pausilipon Children's Hospital Naples Italy; ^8^ Dipartimento di Medicina e Chirurgia Traslazionale Fondazione Policlinico Universitario “A. Gemelli” IRCCS, Catholic University of Sacred Heart Rome Italy

**Keywords:** functional gastrointestinal disorders, pediatric quiescent inflammatory bowel disease, rome IV criteria

## Abstract

**Objectives:**

Pediatric patients with in‐remission inflammatory bowel disease (IBD) often report persistent gastrointestinal symptoms, suggesting a potential overlap with disorders of gut–brain‐interaction (DGBIs). While DGBIs affect up to 40% of the general population, their prevalence in quiescent IBD children remains limited. We aimed to evaluate the prevalence and distribution of DGBIs, based on Rome‐IV criteria, in children with quiescent IBD compared to healthy controls, identifying demographic, clinical and therapeutic factors associated with DGBIs.

**Methods:**

In this multicenter, prospective, controlled study, in‐remission IBD pediatric patients and healthy controls were enrolled completing the Rome IV‐Questionnaire. IBD‐remission was defined by pediatric ulcerative colitis activity index (PUCAI)/pediatric Crohn's disease activity index scores < 10, normal inflammatory markers and recent endoscopic mucosal healing.

**Results:**

Forty‐one pediatric patients with IBD in remission and 179 healthy controls were enrolled. DGBIs were found in 41.46% of IBD‐patients versus 27.93% of controls (*p* = 0.089). Functional Dyspepsia was significantly more prevalent in IBD patients (*p* < 0.001), while irritable bowel syndrome was more frequent among controls, though not significantly (*p* = 0.466). No differences in DGBI prevalence emerged between Crohn's disease and ulcerative colitis (*p* = 0.54). At the multivariate analysis, psychological comorbidities (odds ratio [OR] 40.767, *p* < 0.001) and low weight (0.953, *p* ≤ 0.001) were significantly associated with DGBIs. Notably, 5‐aminosalicylic‐acid (ASA) administration was associated with reduced DGBIs likelihood (OR 0.139, *p* = 0.005).

**Conclusions:**

In our cohort, the overall DGBIs prevalence was numerically but not significantly higher in quiescent‐IBD patients compared to controls, while upper gastrointestinal DGBIs were significantly more frequent in IBD patients. Psychological and nutritional factors emerged as strong predictors of DGBIs, while 5‐ASA may be associated with lower likelihood of DGBIs.

## Abstract (highlights)

**What is Known:**

- Disorders of gut–brain‐interaction (DGBIs) affect up to 40% of the general pediatric population.
- Pediatric patients with quiescent inflammatory bowel disease (IBD) often still report gastrointestinal symptoms.
- Overlap with DGBIs complicates symptom interpretation in IBD.
- Little data exist about DGBIs' prevalence and predictors in quiescent pediatric IBD.

**What is New:**

- In this cohort, children with quiescent IBD showed a numerically but not significantly higher DGBI’ prevalence than controls.
- Psychological comorbidity and low weight emerged as strong predictors of DGBIs.
- Use of 5‐aminosalicylic acid may be associated with lower likelihood of DGBIs.

## 1 INTRODUCTION

The medical management of inflammatory bowel disease (IBD) has evolved over the years thanks to the newly available therapies and the biochemical and endoscopic monitoring of the disease. Several in‐remission IBD patients still complain of gastrointestinal symptoms, suggesting a possible overlap between IBD and disorders of gut–brain interaction (DGBIs), classified and diagnosed according to the Rome IV criteria,^1^ with a worldwide prevalence of about 40% in the general population.^2^ In adult patients with in‐remission IBD, the prevalence of any DGBI has been reported to reach up to 41%,^3^, ^4^ resulting in significantly higher rates in Crohn's disease (CD) than in ulcerative colitis (UC).^5^, ^6^ Regarding the pediatric population, according to a meta‐analysis conducted in 2015, the worldwide prevalence of functional abdominal pain disorders (FAPDs), a subtype of DGBIs including functional dyspepsia, irritable bowel syndrome (IBS), abdominal migraine, and functional abdominal pain not otherwise specified (FAP‐NOS), in children is about 13.5%, with IBS reported as the most frequent disorder (8.8%).^7^ Only a few studies were conducted on pediatric patients to investigate the association between IBD and DGBIs.^8^, ^9^, ^10^, ^11^ A meta‐analysis conducted in 2022 reported an overall prevalence of FAPDs ranging between 9.6% and 29.5% in children with in‐remission IBD, with the overall prevalence of IBS in these patients ranging between 3.9% and 16.1%.^12^ Therefore, despite the differences in criteria used to define quiescent IBD in the included studies, an increased overall prevalence of IBS and FAPDs in children with IBD was described. Nevertheless, none used the current Rome IV criteria to diagnose DGBIs, and only the prevalence of IBS and FAPDs was analyzed. The primary aim of our study was to assess the prevalence of commonly reported DGBIs (functional nausea and vomiting disorders, FAPDs, functional defecation disorders) in pediatric patients with quiescent IBD, compared to a control group of healthy children. Second, we aimed to investigate the presence of any other factors associated with the presence of DGBIs in our population, regardless of the IBD status.

## 2 METHODS

### 2.1 Ethics statement

All patients, healthy subjects, and their parents expressed their written informed consent to participate in the study. The local “Lazio Area 3” Ethical Committee approved the study (Prot IBD_IBS, ID 7277), which was conducted according to the Declaration of Helsinki and the principles of good clinical practice.

### 2.2 Study design

We performed a multicentric, prospective, controlled study in pediatric patients with quiescent IBD recruited during their routine control visit at the Outpatients Pediatric Gastrointestinal Unit of three Italian referral centers in Rome, Italy (Fondazione Policlinico Universitario A. Gemelli IRCCS, Sapienza University of Rome—Sant'Andrea University Hospital, Bambino Gesù Children Hospital). A control group of subjects recruited from the Outpatient Unit of General Pediatrics of Fondazione Policlinico Universitario A. Gemelli IRCCS was enrolled for comparison purposes. All the included children underwent the Rome IV validated questionnaire (QPGS‐RIV) to diagnose DGBIs. Data regarding ongoing maintenance therapy, recent endoscopic evaluation, and recent blood and fecal inflammatory markers were collected among the IBD group.

### 2.3 Study patients

From December 2024 to September 2025, we prospectively enrolled subjects aged 4‐18 years, divided into two groups:

A. In‐remission IBD patients with a previous diagnosis established according to the revised Porto Criteria.^13^ Inclusion required evidence of concomitant clinical, biochemical and endoscopic remission. Clinical remission was defined as pediatric ulcerative colitis activity index/ pediatric Crohn's disease activity index< 10, while biochemical remission was defined as CRP < 0.5 mg/mL and fecal calprotectin <100 μg/g. Endoscopic evaluation was mandatory within the previous 6 months, with endoscopic remission defined as CDEIS < 6 for CD and Mayo score ≤1 for UC.^14^ All of them completed the Rome IV Questionnaire (QPGS‐RIV).
B. At the general pediatrics outpatients unit, healthy subjects were followed for periodic health and auxologic assessment. Those complaining of gastrointestinal issues were asked to complete the QPGS‐RIV.

In both groups, psychological comorbidity was assessed at enrolment by using the Hospital Anxiety and Depression Scale (HADS),^15^ validated in adolescents showing good discriminative validity.^16^ Cut‐off scores were >9 for possible anxiety and >7 for possible depression.^16^

Exclusion criteria were diabetes (type I and II), thyroid disease, major abdominal surgery in the previous 2 years, connective tissue disease, and ongoing corticosteroid or antibiotic therapy.

### 2.4 Statistical analysis

Continuous variables were summarized as median and interquartile range (IQR), while categorical and ordinal variables were expressed as median and range. Between‐group comparisons for non‐parametric variables were conducted using the Mann–Whitney *U* and Chi‐square test. Univariate and multivariate logistic regressions were performed to assess the demographic, clinical and pathological characteristics associated with specific DGBIs diagnosis. The results were reported as odds ratios (ORs) with 95% confidence intervals (95% CIs). An OR with the entire 95% CI less than 1 indicated that the covariate reduced the probability of a DGBI diagnosis. Conversely, when the OR and entire 95% CI were greater than 1, the covariate increased the abovementioned probability. An OR with a 95% CI equal to 1 indicated that the covariate did not significantly influence this probability. For all comparisons, two‐sided *p*‐values < 0.05 were considered statistically significant. Statistical analyses were performed using STATA software version n. 18.

## 3 RESULTS

### 3.1 Demographics and clinical evaluations

**Figure 1:**
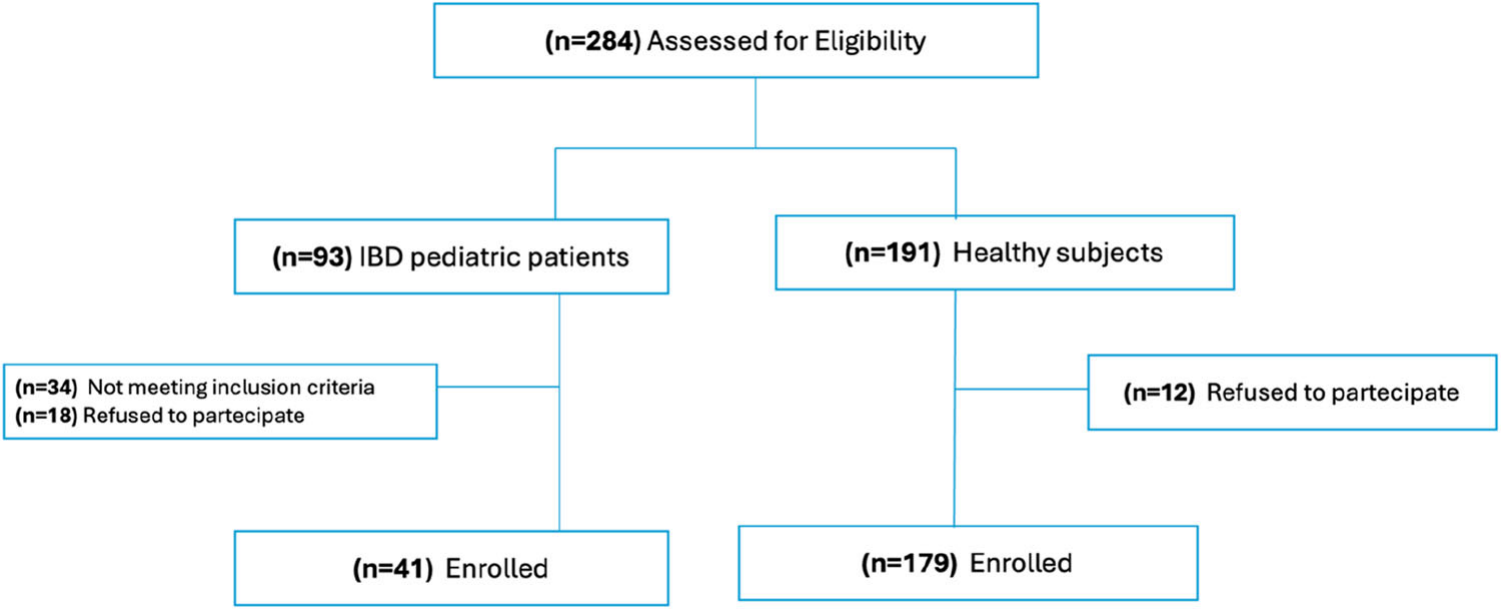
Consort diagram of the study. IBD, inflammatory bowel disease.

From January 2024 to January 2025, we prospectively screened 93 IBD children and 191 healthy subjects. Of these, 52 IBD patients and 12 healthy subjects were excluded for not meeting the inclusion criteria or refusing to participate (Figure 1).

Forty‐one IBD pediatric patients (41.5% females) were enrolled after the screening phase. The control group included 179 children, with a female representation of 68.2%. The median age of participants in the IBD group was 17 years (IQR 14–17), while the median age in the healthy control group was 15 years (IQR 11–17). The IBD cohort consisted of 17 patients with CD and 24 with UC. The two subgroups were comparable in terms of demographic characteristics, with a female prevalence of 52.94% in the CD group vs 33.33% in the UC group (*p* = 0.209), and a median age of 16 years (IQR 14‐17) in the CD group versus 17 years (IQR 14.5–17) in the UC group (*p* = 0.494) (Supporting Information S1: Table S1).

Demographic characteristics of the study population are shown in Table 1.

**Table 1:** Demographic characteristics of the study population.

	IBD in remission	General population	*p*
Total	41	179	
Female, *N* (%)	17 (41.5)	122 (68.2)	0.001
Median age, years (IQR)	17 (14–17)	15 (11–17)	0.066
Weight, kg (IQR)	56 (49.7–65)	53 (37–62)	0.013
Height, cm (IQR)	166.5 (158–174)	162 (138.5–170)	0.018
BMI percentile, % (IQR)	44.5 (21.6–62)	60 (35–76)	0.158
IBD subclassification			
Ulcerative colitis (%)	24 (58.5)		
Crohn disease (%)	17 (41.5)		

Abbreviations: BMI, body mass index; IBD, inflammatory bowel disease; IQR, interquartile range.

### 3.2 DGBI's prevalence in the study cohort

Table 2 provides an overview of the distribution of DGBIs in our population. No statistically significant difference was observed in the overall prevalence of DGBIs between IBD pediatric patients and the general population (41.46% vs. 27.93%, respectively; *p* = 0.089). Although the difference did not reach statistical significance, a numerically higher prevalence of DGBIs was noted among IBD patients. Considering the differences in baseline demographic characteristics between groups, a sub‐analysis adjusted for sex and age was performed, and the prevalence of DGBIs remained higher in the IBD group after this adjustment (Supporting Information S1: Tables S2–S5).

**Table 2:** DGBI distribution in IBD patients and the general population.

	IBD in remission	General population	*p*
Total	41	179	
DGBI (%)	17 (41.46)	50 (27.93)	0.089
Overlap (%)	9 (21.95)	36 (20.11)	0.792
Functional dyspepsia *N* (%)	10 (24.36)	7 (3.91)	<0.001
Postprandial dystress syndrome (%)	7 (17.07)	5 (2.79)	<0.001
Epigastric pain syndrome (%)	5 (12.20)	3 (1.68)	0.001
Abdominal migraine (%)	0	0	‐
Cyclic vomiting syndrome (%)	0	2 (1.12)	0.497
Functional nausea and vomiting (%)	3 (7.32)	3 (1.68)	0.045
Rumination syndrome (%)	2 (4.88)	3 (1.68)	0.215
Aerophagia (%)	4 (9.76)	4 (2.23)	0.02
Irritable bowel syndrome (%)	6 (14.63)	35 (19.55)	0.466
Functional abdominal pain (%)	1 (2.44)	20 (11.17)	0.086
Functional constipation (%)	6 (14.63)	22 (12.29)	0.685
Nonretentive fecal incontinence (%)	1 (2.44)	2 (1.12)	0.51

Abbreviations: DGBI, disorders of gut–brain‐interaction; IBD, inflammatory bowel disease.

A more detailed analysis of individual DGBI subtypes revealed a significantly higher prevalence of upper gastrointestinal DGBIs in the IBD group compared to the general population. In particular, functional dyspepsia was significantly more frequent among IBD patients versus controls (24.36% vs. 3.91% respectively, *p* < 0.001), including both postprandial distress syndrome (PDS, 17.07% vs 2.79%, *p* < 0.001) and epigastric pain syndrome (EPS, 12.20% vs. 1.68%, *p* = 0.001) subtypes. In contrast, no significant differences were found for other upper gastrointestinal DGBIs, such as functional nausea and vomiting, rumination syndrome, aerophagia, or cyclic vomiting syndrome. Conversely, lower gastrointestinal DGBIs, including irritable bowel syndrome (IBS, 14.63% in IBD patients vs. 19.55% in controls, *p* = 0.466) and functional abdominal pain not otherwise specified (FAP‐NOS, 2.44% vs. 11.17%, *p* = 0.086), were more frequently reported in the control group. However, these differences did not reach statistical significance. Similarly, no significant difference was found in the prevalence of functional defecation disorders between the two groups (*p* > 0.05) (Table 2).

DGBI overlap was defined as the concomitant presence of more than one DGBI in the same patient. In our cohort, no significant differences were found in the prevalence of DGBI overlap between the IBD group and controls (21.95% vs. 20.11% respectively, *p* = 0.792) (Table 2).

Furthermore, our data did not reveal any significant difference in the prevalence of DGBIs between patients with CD and those with UC (47.06% vs. 37.5%, *p* = 0.540). Moreover, no specific DGBI subtype appeared to be predominantly associated with either subgroup, suggesting a homogeneous distribution of functional gastrointestinal symptoms across different IBD phenotypes in our pediatric cohort (Supporting Information S1: Table S1).

### 3.3 Factors associated with DGBI's diagnosis

Univariate and multivariate predictors of DGBI diagnosis in the enrolled population are shown in Table 3. Notably, the presence of psychological comorbidities emerged as the strongest independent predictor, with an OR of 37.97 (95% CI: 16.66–86.56; *p* < 0.001). In addition, lower body weight was associated with higher odds of having a DGBI, showing significant associations in both univariate (OR 0.973, 95% CI: 0.956–0.994, *p* = 0.001) and multivariate analyses (OR 0.953, 95% CI: 0.93–0.978, *p* < 0.001). Undernutrition, defined as BMI centile <3rd, was also significantly associated with a higher likelihood of DGBIs in univariate analysis (OR 4.93, 95% CI: 1.20–20.37; *p* = 0.027). Younger age, higher BMI percentile, and lower weight and height were all significantly associated with an increased risk of DGBIs, while sex and overall IBD status did not show significant associations. Furthermore, the presence of IBD in general (OR 1.83, *p* = 0.092) or specifically CD (OR 1.48, *p* = 0.541) was not significantly associated with DGBIs.

**Table 3:** Predictors of DGBI in the enrolled population (*n* = 220): Univariate and multivariate analysis.

	Odds ratio (95% CI)	*p*‐value	Odds ratio (95% CI)	*p*‐value
Female	0.564 (0.313–1.01)	0.056		
Age	0.881 (0.956–0.989)	<0.001		
Weight	0.973 (0.956–0.994)	0.001	0.953 (0.93–0.978)	<0.001
Height	0.983 (0.972–0.994)	0.002		
BMI	0.912 (0.821–1.012)	0.081		
BMI percentile	1.019 (1.006–1.031)	0.003		
Undernutrition	4.933 (1.195–20.369)	0.027		
IBD	1.828 (0.906–3.687)	0.092		
Crohn's disease	1.481 (0.42–5.228)	0.541		
Psychological comorbidity	37.968 (16.655–86.557)	<0.001	40.767 (14.714–112.953)	<0.001

Abbreviations: BMI, body mass index; CI, confidence interval; DGBI, disorders of gut–brain‐interaction; IBD, inflammatory bowel disease.

Evaluating specific diagnoses, both FD and IBS were strongly associated with psychological comorbidities in the multivariate analysis (OR 3.267, *p* = 0.021 and OR 12.005, *p* < 0.001, respectively). FD was also significantly associated with IBD (OR 11.481, *p* < 0.001) and IBS (OR 4.516, *p* = 0.027). At the same time, IBS showed strong associations with FD (OR 42.681, *p* < 0.001) in the multivariate analysis and with undernutrition (OR 9.886, *p* = 0.002) in the univariate model. Demographic variables such as sex and age were not significantly associated with FD or IBS.

Furthermore, data about the ongoing maintenance therapy were analyzed in the IBD group (Supporting Information S1: Table S6). Univariate analysis evaluating the association between IBD‐related therapies and the presence of DGBIs revealed that most treatments (such as probiotics, azathioprine, infliximab, adalimumab, vedolizumab, or elemental nutrition) did not significantly influence DGBI occurrence. Nevertheless, 5‐aminosalicylic acid (ASA) was associated with a significantly lower risk of DGBIs (OR 0.139; 95% CI: 0.034–0.56; *p* = 0.005), suggesting a potential protective effect. In subgroup analyses, 5‐ASA showed a significant inverse association with DGBI in UC but not in CD, although results in CD were limited by the small sample size (Supporting Information S1: Tables S7 and S8).

Finally, Supporting Information S1: Table 9 summarizes the univariate and multivariate analyses of factors associated with DGBI overlap. Psychological comorbidity emerged as the strongest independent predictor of DGBI overlap, both in the univariate (OR 35.88, *p* < 0.001) and multivariate (OR = 10.99, *p* < 0.001) model. Additionally, at the univariate analysis, undernutrition (OR = 8.67, *p* = 0.003) and higher BMI percentile (OR = 1.023, *p* = 0.002) were significantly associated with an increased likelihood of overlap. Conversely, no significant association was found between IBD status or CD subtype and the risk of DGBI overlap.

## 4 DISCUSSION

In this multicenter prospective study, we aimed to assess the prevalence of DGBIs in pediatric patients with quiescent IBD, using the Rome IV diagnostic criteria. Our data revealed a slightly higher prevalence of DGBIs in IBD patients compared to controls, mainly related to upper gastrointestinal functional disorders and strongly associated with psychological and nutritional factors.

Although the overall prevalence of DGBIs was higher in the IBD group than healthy controls (41.46% vs. 27.93%, respectively), this difference did not reach statistical significance (*p* = 0.089). However, this trend is consistent with previous literature reporting DGBI prevalence up to 41% in adult patients with IBD in remission.^3^ When analyzing the distribution of DGBI subtypes within our cohort, the prevalence of IBS among pediatric patients with quiescent IBD was 14.63%, aligning with previously published data reporting a prevalence that ranged between 3.90% and 16.10% in this population.^12^

In our cohort, a significantly higher prevalence of upper gastrointestinal DGBIs was observed in the IBD group compared to controls, particularly functional dyspepsia and its subtypes, postprandial distress syndrome, and epigastric pain syndrome. In contrast, lower gastrointestinal symptoms, including IBS and FAP‐NOS, were more frequently reported in the control group, although the differences were not statistically significant. This finding may be partially explained by persistent abnormalities in upper gastrointestinal motility, such as delayed gastric emptying, which have been reported in pediatric patients with CD even during remission, although available data remain limited.^17^ In contrast, in adults with inactive CD, delayed gastric transit has also been linked to upper gastrointestinal symptoms.^18^

In contrast to findings from previous studies conducted in adult populations,^5^, ^6^ our analysis did not reveal any significant differences in the prevalence of DGBIs between CD and UC patients. This lack of distinction may be attributed to our cohort's relatively small sample size. Adult studies suggest that CD and UC may share common pathophysiological mechanisms involving functional and structural alterations in enteric neurons and ganglia, which may disrupt microbiota–gut–brain axis signaling, potentially contributing to the persistence or exacerbation of gastrointestinal symptoms, even during clinical remission in both IBD subtypes.^19^ Nevertheless, pediatric‐specific data on the pathophysiology of DGBIs in quiescent IBD are still limited and warrant further investigation.

Notably, psychological comorbidities emerged as the strongest independent predictor of DGBIs in our cohort, underlying the pivotal role of the microbiota‐gut–brain axis in the pathophysiology of functional gastrointestinal symptoms.^20^ The interaction between psychological factors and gastrointestinal function is well established, with stress influencing gut microbiota composition and consequently gut motility, visceral hypersensitivity, and mucosal immune activity.^21^ These findings highlight the need for a multidisciplinary approach in the management of children with DGBIs, emphasizing the importance of incorporating psychological screening and support into routine care for both IBD and non‐IBD patients presenting with functional symptoms.

DGBI overlap represents a clinically relevant phenotype associated with more than one DGBI in the same individual. In our cohort, psychological comorbidity and undernutrition were independently associated with an increased risk of overlap, while neither IBD nor CD subtype significantly influenced this risk. These findings suggest that although organic diseases may be initial trigger by significantly modifying gut microbiota and altering gut barrier, DGBI overlap are sustained by functional and psychosocial factors. Several mechanisms may explain this data. Visceral hypersensitivity seems to play a pivotal role, with evidence that patients with DGBI overlap may experience a more diffuse form of visceral hyperalgesia.^22^ Additionally, altered gastrointestinal motility described in these patients, such as delayed gastric emptying, may further contribute to the symptoms’ presentation.^23^ Finally, emerging evidence highlights the potential contribution of gut microbiota dysbiosis in the pathogenesis of DGBI overlap, reinforcing the great importance of microbiota in the pathogenesis of these conditions.^24^ However, most supporting evidence derives from adult populations, while pediatric data remain limited. Therefore, these mechanisms should be considered speculative.

In our study, both undernutrition, defined as a BMI percentile <3rd, and overnutrition, represented by higher BMI percentiles, were significantly associated with a greater prevalence of DGBIs and DGBI overlap. These findings align with previous evidence in adult populations reporting a significantly higher prevalence of DGBIs in obese adults compared to normal or overweight individuals (44.2% vs. 39.6%; OR = 1.20, 95% CI: 1.12–1.30).^25^ Conversely, the observed link between low weight and increased DGBI prevalence may reflect the complex bidirectional relationship between nutritional status and gastrointestinal function. Children with DGBIs often exhibit reduced appetite, altered eating behaviors, or dietary restrictions due to symptom‐related food avoidance, which may contribute to undernutrition. Additionally, undernutrition itself may exacerbate gut dysbiosis and impair mucosal integrity, potentially playing a role in the pathophysiology of DGBIs.^26^

An intriguing and potentially clinically relevant finding of our study was that 5‐aminosalicylic acid (5‐ASA) therapy was more commonly observed in patients with no DGBIs, suggesting a potential association with a lower risk of developing DGBIs. Although this observation should be interpreted cautiously, as it may reflect confounding factors, some evidence from the literature suggests that mesalamine may modestly improve functional gastrointestinal symptoms. A randomized, double‐blind, placebo‐controlled trial demonstrated that mesalamine significantly reduced colonic mast cell infiltration in patients with IBS and improved overall well‐being despite not achieving significant changes in pain or bowel habits.^27^ More recently, a meta‐analysis of eight randomized controlled trials (RCTs) involving 820 IBS patients found that mesalamine was modestly more effective than placebo in improving global IBS symptoms (RR 0.86, 95% CI: 0.79–0.95; NNT = 10), especially in patients with diarrhea‐predominant IBS, although the overall quality of evidence was low.^28^

These data suggest a possible immune‐mediated or microbiota‐modulating mechanism through which mesalamine may influence gut‐brain axis function, but it remains uncertain whether 5‐ASA could influence DGBI development in quiescent IBD patients, highlighting the need for further investigations in larger controlled studies.

A major strength of this manuscript is that it represents the first large, prospective, controlled, multicenter study assessing the prevalence of DGBIs, as defined by the Rome IV criteria, in a pediatric population with quiescent IBD. The study population was rigorously selected using stringent criteria to define quiescent IBD. Furthermore, a wide range of DGBIs was evaluated, including the assessment of DGBI overlap, alongside a thorough evaluation of key comorbidities relevant to DGBI, such as nutritional status and psychological comorbidities. However, some limitations must be acknowledged, such as the small sample size of the IBD cohort and the uneven sex distribution between the two groups, with a higher proportion of females among controls, representing a potential confounding factor, partially explaining why IBS and Functional abdominal pain rates were slightly higher in the control group. A further limitation of our study is that the QPGS‐RIV was administered only to symptomatic controls, while asymptomatic children were assumed to be free of DGBIs, introducing a potential selection bias.

## 5 CONCLUSION

In conclusion, our findings highlight the high prevalence of DGBIs among pediatric patients with quiescent IBD, with predominant upper gastrointestinal manifestations and a strong association with psychological comorbidities. Of particular interest, mesalamine therapy was associated with a significantly lower risk of developing DGBIs. However, this finding should be interpreted with caution and warrants further investigation. Future prospective studies are needed to better understand the natural history of DGBIs in this population and to develop more targeted treatment strategies.

## CONFLICT OF INTEREST STATEMENT

The authors declare no conflicts of interest.

## Supporting information

New Supplementary, JPGN.

## Data Availability

The data supporting this study's findings are available from the corresponding author upon reasonable request.
