# Supplementary material for: Prevalence of disorders of gut–brain‐interaction in pediatric patients with in‐remission inflammatory bowel disease: An Italian multicenter study
Source: J Pediatr Gastroenterol Nutr. 2026 Feb 23;82(5):1171–7. doi: 10.1002/jpn3.70386 (PMC13150794; doi:10.1002/jpn3.70386)
Supplement: Supplementary file 1 — New Supplementary, JPGN. [file JPN3-82-1171-s001.docx]

**Supplementary Table 1**: Descriptive Comparison Between Crohn’s Disease and Ulcerative Colitis Patients

|  | Crohn’s Disease | Ulcerative Colitis | | p |
| --- | --- | --- | --- | --- |
|  |  |  |  | |
| Total | 17 | 24 |  | |
| Median age, years (iqr) | 16 (14-17) | 17 (14.5-17) | 0.494 | |
| Female, n (%) | 9 (52.94) | 8 (33.33) | 0.209 | |
| Weight, kg (iqr) | 53 (46.6-65) | 57.8 (52-65) | 0.169 | |
| Height, cm (iqr) | 164 (154-170) | 167 (160-176) | 0.226 | |
| Bmi, kg/m2 (iqr) | 19.5 (18.13-21) | 21 (19.2-24) | 0.103 | |
| Bmi centile, % (iqr) | 31 (15-52) | 50 (25-63.4) | 0.169 | |
| Dgbi (%) | 8 (47.06) | 9 (37.5) | 0.540 | |
| Overlap (%) | 3 (17.65) | 6 (25) | 0.575 | |
| Aerophagia (%) | 2 (11.76) | 2 (8.33) | 0.715 | |
| Irritable Bowel Syndrome (%) | 2 (11.76) | 4 (16.67) | 0.662 | |
| Functional Dyspepsia (%) | 5 (29.41) | 5 (20.83) | 0.529 | |
| Postprandial Dystress Syndrome (%) | 3 (17.65) | 4 (16.67) | 0.934 | |
| Epigastric Pain Syndrome (%) | 3 (17.65) | 2 (8.33) | 0.369 | |
| Abdominal Migraine (%) | 0 | 0 |  | |
| Functional Abdominal Pain (%) | 0 | 1 (4.17) | 0.394 | |
| Cyclic Vomiting Syndrome (%) | 0 | 0 |  | |
| Functional Nausea and Vomiting (%) | 1 (5.88) | 2 (8.33) | 0.767 | |
| Rumination Syndrome (%) | 1 (5.88) | 1 (4.17) | 0.802 | |
| Functional Constipation (%) | 3 (17.65) | 3 (12.5) | 0.646 | |
| Nonretentive Fecal Incontinence (%) | 0 | 1 (4.17) | 0.394 | |
| Psychological Comorbidity (%) | 6 (35.29) | 15 (62.5) | 0.086 | |
| Upper GI Endoscopy (%) | 16 (94.12) | 9 (37.5) | **<0.001** | |
| Focally Enhanced Gastritis (%) | 10 (58.82) | 4 (16.67) | **0.005** | |

**Supplementary Table 2:** Descriptive table in the female population

| Variable | IBD in remission (n = 17) | General population (n=122) | p-value |
| --- | --- | --- | --- |
| Age (years) | 17.0 (13.0-17.0) | 16.0 (14.0-17.0) | 0.993 |
| BMI (Kg/m^2^) | 18.9 (17.9-21.0) | 20.0 (18.8-21.0) | 0.220 |
| BMI Percentile (%) | 32.5 (15.5-50.0) | 50.0 (30.0-74.5) | 0.025 |
| Malnutrition (%) | 0 (0.0%) | 3 (2.5%) | 0.526 |
| IBD subclassification |  |  |  |
| Ulcerative colitis (%) | 8 (47.1%) | 0 (0%) |  |
| Crohn's disease (%) | 9 (52.9%) | 0 (0%) |  |
| DGBI (%) | 10 (58.8%) | 26 (21.3%) | <0.001 |
| Overlap (%) | 5 (29.4%) | 21 (17.2%) | 0.227 |
| Irritable bowel syndrome (%) | 5 (29.4%) | 19 (15.6%) | 0.157 |
| Functional dyspepsia (%) | 8 (47.1%) | 3 (2.5%) | <0.001 |
| Postprandial distress syndrome (%) | 5 (29.4%) | 3 (2.5%) | <0.001 |
| Epigastric pain syndrome (%) | 4 (23.5%) | 1 (0.8%) | <0.001 |
| Abdominal Migraine (%) | 0 (0.0%) |  | NA |
| Functional abdominal pain (%) | 0 (0.0%) | 12 (9.8%) | 0.176 |
| Cyclic vomiting syndrome (%) | 0 (0.0%) | 2 (1.6%) | 0.595 |
| Functional nausea and vomiting (%) | 2 (11.8%) | 3 (2.5%) | 0.054 |
| Rumination syndrome (%) | 0 (0.0%) | 2 (1.6%) | 0.595 |
| Aerophagia (%) | 2 (11.8%) | 2 (1.6%) | 0.019 |
| Functional constipation (%) | 2 (11.8%) | 10 (8.2%) | 0.624 |
| Nonretentive fecal incontinence (%) | 1 (5.9%) | 0 (0.0%) | 0.007 |
| Psychological comorbidity (%) | 7 (41.2%) | 19 (15.6%) | 0.011 |
| Therapy |  |  |  |
| Probiotic (%) | 5 (29.4%) | 11 (9.0%) | 0.014 |
| ASA (%) | 9 (52.9%) | 0 (0.0%) | <0.001 |
| Azathioprine (%) | 3 (17.6%) | 0 (0.0%) | <0.001 |
| Infliximab (%) | 3 (17.6%) | 0 (0.0%) | <0.001 |
| Adalimumab (%) | 5 (29.4%) | 0 (0.0%) | <0.001 |
| Vedolizumab (%) | 1 (5.9%) | 0 (0.0%) | 0.007 |
| Modulen (%) | 1 (5.9%) | 0 (0.0%) | 0.007 |
| Colestiramina (%) | 0 (0.0%) | 3 (2.5%) | 0.513 |
| Macrogol (%) | 1 (5.9%) | 13 (10.7%) | 0.540 |
| Antispastic (%) | 0 (0.0%) | 7 (5.7%) | 0.311 |
| Antiemetic (%) | 0 (0.0%) | 12 (9.8%) | 0.176 |
| Prokinetics (%) | 0 (0.0%) | 3 (2.5%) | 0.513 |

**Supplementary table 3:** Descriptive table in the male population

| Variable | IBD in remission  (n = 24) | General population (n=57) | p-value |
| --- | --- | --- | --- |
| Age (years) | 16.5 (14.5–17.0) | 12.0 (6.0–16.0) | <0.001 |
| BMI (Kg/m^2^) | 20.8 (19.5–24.0) | 19.0 (17.7–21.0) | 0.004 |
| BMI Percentile (%) | 52.8 (25.0–63.0) | 70.0 (50.0–80.0) | 0.013 |
| Malnutrition (%) | 0 (0.0%) | 6 (10.5%) | 0.113 |
| IBD subclassification |  |  | NA |
| Ulcerative colitis (%) | 8 (33.3%) | 0 (0.0%) |  |
| Crohn's disease (%) | 16 (66.7%) | 0 (0.0%) |  |
| DGBI (%) | 7 (29.2%) | 24 (42.1%) | 0.274 |
| Overlap (%) | 4 (16.7%) | 15 (26.3%) | 0.349 |
| Irritable bowel syndrome (%) | 1 (4.2%) | 16 (28.1%) | 0.016 |
| Functional dyspepsia (%) | 2 (8.3%) | 4 (7.0%) | 0.836 |
| Postprandial distress syndrome  (%) | 2 (8.3%) | 2 (3.5%) | 0.360 |
| Epigastric pain  syndrome (%) | 1 (4.2%) | 2 (3.5%) | 0.886 |
| Abdominal Migraine (%) | 0 (0.0%) | 0 (0.0%) | NA |
| Functional abdominal pain (%) | 1 (4.2%) | 8 (14.0%) | 0.197 |
| Cyclic vomiting syndrome (%) | 0 (0.0%) | 0 (0.0%) | NA |
| Functional nausea and vomiting (%) | 1 (4.2%) | 0 (0.0%) | 0.121 |
| Rumination syndrome (%) | 2 (8.3%) | 1 (1.8%) | 0.152 |
| Aerophagia (%) | 2 (8.3%) | 2 (3.5%) | 0.360 |
| Functional constipation (%) | 4 (16.7%) | 12 (21.1%) | 0.651 |
| Nonretentive fecal incontinence (%) | 0 (0.0%) | 2 (3.5%) | 0.353 |
| Psychological comorbidity (%) | 14 (58.3%) | 21 (36.8%) | 0.075 |
| Therapy |  |  |  |
| Probiotic (%) | 4 (16.7%) | 11 (19.3%) | 0.781 |
| ASA (%) | 14 (58.3%) | 0 (0.0%) | <0.001 |
| Azathioprine (%) | 6 (25.0%) | 0 (0.0%) | <0.001 |
| Infliximab (%) | 11 (45.8%) | 0 (0.0%) | <0.001 |
| Adalimumab (%) | 5 (20.8%) | 0 (0.0%) | <0.001 |
| Vedolizumab (%) | 1 (4.2%) | 0 (0.0%) | 0.121 |
| Modulen (%) | 4 (16.7%) | 0 (0.0%) | 0.002 |
| Colestiramina (%) | 1 (4.2%) | 4 (7.0%) | 0.626 |
| Macrogol (%) | 1 (4.2%) | 12 (21.1%) | 0.059 |
| Antispastic (%) | 0 (0.0%) | 10 (17.5%) | 0.028 |
| Antiemetic (%) | 0 (0.0%) | 1 (1.8%) | 0.514 |
| Prokinetics (%) | 0 (0.0%) | 5 (8.8%) | 0.134 |

**Supplementary table 4**: Descriptive table in patients aged <15 years

| Variable | IBD in remission (n = 24) | General population (n=57) | p-value |
| --- | --- | --- | --- |
| Age (years) | 13.0 (12.0–14.0) | 9.0 (6.0–13.0) | 0.003 |
| BMI (Kg/m^2^) | 19.0 (17.6–20.0) | 18.0 (16.3–19.8) | 0.400 |
| BMI Percentile (%) | 48.5 (31.0–54.0) | 70.0 (35.0–83.0) | 0.051 |
| Female sex (%) | 5 (45.5%) | 34 (47.9%) | 0.880 |
| Malnutrition (%) | 0 (0.0%) | 8 (11.3%) | 0.264 |
| IBD subclassification |  |  |  |
| Ulcerative colitis (%) | 5 (45.5%) | 0 (0.0%) |  |
| Crohn's disease (%) | 6 (54.5%) | 0 (0.0%) |  |
| DGBI (%) | 6 (54.5%) | 36 (50.7%) | 0.813 |
| Overlap (%) | 2 (18.2%) | 26 (36.6%) | 0.230 |
| Irritable bowel syndrome (%) | 2 (18.2%) | 26 (36.6%) | 0.230 |
| Functional dyspepsia (%) | 1 (9.1%) | 5 (7.0%) | 0.808 |
| Postprandial distress syndrome (%) | 1 (9.1%) | 2 (2.8%) | 0.302 |
| Epigastric pain  syndrome (%) | 1 (9.1%) | 3 (4.2%) | 0.486 |
| Abdominal Migraine (%) | 0 (0.0%) | 0 (0.0%) | NA |
| Functional abdominal pain (%) | 0 (0.0%) | 16 (22.5%) | 0.079 |
| Cyclic vomiting syndrome (%) | 0 (0.0%) | 0 (0.0%) | NA |
| Functional nausea and vomiting (%) | 0 (0.0%) | 1 (1.4%) | 0.692 |
| Rumination syndrome (%) | 1 (9.1%) | 3 (4.2%) | 0.486 |
| Aerophagia (%) | 2 (18.2%) | 3 (4.2%) | 0.072 |
| Functional constipation (%) | 3 (27.3%) | 18 (25.4%) | 0.892 |
| Nonretentive fecal incontinence (%) | 0 (0.0%) | 0 (0.0%) | NA |
| Psychological comorbidity (%) | 5 (45.5%) | 26 (36.6%) | 0.574 |
| Therapy |  |  |  |
| Probiotic (%) | 0 (0.0%) | 14 (19.7%) | 0.106 |
| ASA (%) | 5 (45.5%) | 0 (0.0%) | <0.001 |
| Azathioprine (%) | 4 (36.4%) | 0 (0.0%) | <0.001 |
| Infliximab (%) | 2 (18.2%) | 0 (0.0%) | <0.001 |
| Adalimumab (%) | 3 (27.3%) | 0 (0.0%) | <0.001 |
| Vedolizumab (%) | 0 (0.0%) | 0 (0.0%) | NA |
| Modulen (%) | 1 (9.1%) | 0 (0.0%) | 0.011 |
| Colestiramina (%) | 0 (0.0%) | 6 (8.5%) | 0.317 |
| Macrogol (%) | 1 (9.1%) | 21 (29.6%) | 0.154 |
| Antispastic (%) | 0 (0.0%) | 12 (16.9%) | 0.140 |
| Antiemetic (%) | 0 (0.0%) | 1 (1.4%) | 0.692 |
| Prokinetics (%) | 0 (0.0%) | 13 (18.3%) | 0.122 |

**Supplementary table 5:** Descriptive table the patients aged ≥ 15 years

| Variable | IBD in remission  (n = 30) | General population  (n= 108) | p-value |
| --- | --- | --- | --- |
| Age (years) | 17.0 (16.0–17.0) | 17.0 (16.0–18.0) | 0.914 |
| BMI (Kg/m^2^) | 20.8 (19.4–23.5) | 20.0 (19.0–21.7) | 0.474 |
| BMI Percentile (%) | 41.0 (16.0–63.2) | 50.0 (35.0–70.0) | 0.078 |
| Female sex (%) | 12 (40.0%) | 88 (81.5%) | <0.001 |
| Malnutrition (%) | 0 (0.0%) | 1 (0.9%) | 0.609 |
| IBD subclassification |  |  |  |
| Ulcerative colitis (%) | 18 (60.0%) | 0 (0.0%) | NA |
| Crohn's disease (%) | 12 (40.0%) | 0 (0.0%) | NA |
| DGBI (%) | 11 (36.7%) | 14 (13.0%) | 0.003 |
| Overlap (%) | 7 (23.3%) | 10 (9.3%) | 0.038 |
| Irritable bowel syndrome (%) | 4 (13.3%) | 9 (8.3%) | 0.407 |
| Functional dyspepsia (%) | 9 (30.0%) | 2 (1.9%) | <0.001 |
| Postprandial distress syndrome  (%) | 6 (20.0%) | 3 (2.8%) | <0.001 |
| Epigastric pain  syndrome (%) | 1 (3.3%) | 0 (0.0%) | 0.057 |
| Abdominal Migraine (%) | 0 (0.0%) | 0 (0.0%) | NA |
| Functional abdominal pain (%) | 1 (3.3%) | 4 (3.7%) | 0.923 |
| Cyclic vomiting syndrome (%) | 0 (0.0%) | 2 (1.9%) | 0.453 |
| Functional nausea and vomiting (%) | 3 (10.0%) | 2 (1.9%) | 0.035 |
| Rumination syndrome (%) | 1 (3.3%) | 0 (0.0%) | 0.057 |
| Aerophagia (%) | 2 (6.7%) | 1 (0.9%) | 0.056 |
| Functional constipation (%) | 3 (10.0%) | 4 (3.7%) | 0.164 |
| Nonretentive fecal incontinence (%) | 1 (3.3%) | 2 (1.9%) | 0.623 |
| Psychological comorbidity (%) | 16 (53.3%) | 14 (13.0%) | <0.001 |
| Therapy |  |  |  |
| Probiotic (%) | 9 (30.0%) | 8 (7.4%) | <0.001 |
| ASA (%) | 18 (60.0%) | 0 (0.0%) | <0.001 |
| Azathioprine (%) | 5 (16.7%) | 0 (0.0%) | <0.001 |
| Infliximab (%) | 12 (40.0%) | 0 (0.0%) | <0.001 |
| Adalimumab (%) | 7 (23.3%) | 0 (0.0%) | <0.001 |
| Vedolizumab (%) | 2 (6.7%) | 0 (0.0%) | 0.007 |
| Modulen (%) | 4 (13.3%) | 0 (0.0%) | <0.001 |
| Colestiramina (%) | 1 (3.3%) | 1 (0.9%) | 0.329 |
| Macrogol (%) | 1 (3.3%) | 4 (3.7%) | 0.923 |
| Antispastic (%) | 0 (0.0%) | 5 (4.6%) | 0.230 |
| Antiemetic (%) | 0 (0.0%) | 3 (2.8%) | 0.356 |
| Prokinetics (%) | 0 (0.0%) | 4 (3.7%) | 0.285 |

**Supplementary Table 6**: Univariate Analysis of Factors Associated with DGBI in the IBD Population (n = 41)

|  | Odds Ratio (95% CI) | P-value |
| --- | --- | --- |
| Female | 3.469 (0.939 – 12.817) | 0.062 |
| Age | 0.937 (0.695 - 1.264) | 0.671 |
| Weight | 0.949 (0.894 – 1.007) | 0.083 |
| Height | 0.937 (0.873 – 1.007) | 0.075 |
| BMI | 0.876 (0.707 – 1.086) | 0.227 |
| BMI percentile | 0.993 (0.968 – 1.019) | 0.616 |
| Crohn's disease | 1.481 (0.42 - 5.228) | 0.541 |
| Psychological comorbidity | 2.567 (0.711 – 9.267) | 0.15 |
| Therapy |  |  |
| Probiotic | 2.083 (0.466 – 9.308) | 0.337 |
| ASA | 0.139 (0.034 - 0.56) | **0.005** |
| Azathioprine | 2.083 (0.466 – 9.308) | 0.337 |
| Infliximab | 0.694 (0.183 - 2.628) | 0.591 |
| Adalimumab | 0.923 (0.216 – 3.944) | 0.914 |
| Vedolizumab | 1.0 |  |
| Modulen | 0.313 (0.032 – 3.079) | 0.319 |

**Supplementary Table 7**: Univariate Analysis of Factors Associated with DGBI in the Ulcerative Colitis Population (n = 24)

| Covariate | Odds Ratio (OR) | 95% CI | p value |
| --- | --- | --- | --- |
| Female sex | 5.00 | 0.81 – 31.00 | 0.084 |
| Age (years) | 0.91 | 0.61 – 1.37 | 0.662 |
| BMI (kg/m²) | 0.80 | 0.58 – 1.10 | 0.165 |
| Psychiatric comorbidity | 3.06 | 0.47 – 19.88 | 0.242 |
| Probiotic use | 2.00 | 0.31 – 13.06 | 0.470 |
| ASA therapy | 0.12 | 0.02 – 0.90 | **0.039** |
| Azathioprine | 1.14 | 0.15 – 8.59 | 0.897 |
| Infliximab | 0.57 | 0.10 – 3.18 | 0.524 |
| Adalimumab | 0.81 | 0.06 – 10.48 | 0.875 |

**Supplementary Table 8**: Univariate Analysis of Factors Associated with DGBI in Crohn’s Disease Population (n = 17)

| Covariate | Odds Ratio (OR) | 95% CI | p value |
| --- | --- | --- | --- |
| Female sex | 2.08 | 0.30 – 14.55 | 0.459 |
| Age (years) | 0.98 | 0.62 – 1.55 | 0.945 |
| BMI (kg/m²) | 1.04 | 0.72 – 1.51 | 0.841 |
| Psychiatric comorbidity | 3.50 | 0.43 – 28.45 | 0.241 |
| Probiotic use | 2.67 | 0.19 – 36.76 | 0.464 |
| ASA therapy | 0.11 | 0.01 – 1.36 | 0.086 |
| Azathioprine | 4.80 | 0.38 – 59.89 | 0.223 |
| Infliximab | 1.17 | 0.12 – 10.99 | 0.893 |
| Adalimumab | 0.75 | 0.11 – 5.24 | 0.772 |
| Modulen | 0.18 | 0.02 – 2.12 | 0.172 |

**Supplementary Table 9**: Univariate and Multivariate Analyses of Factors Associated with DGBI Overlap in the Study Population (n = 220)

|  | Odds Ratio (95% CI) | P-value | Odds Ratio (95% CI) | P-value |
| --- | --- | --- | --- | --- |
| Female | 0.751 (0.385 - 1.464) | 0.4 |  |  |
| Age | 0.898 (0.841 - 0.96) | **0.02** |  |  |
| Weight | 0.978 (0.96 – 0.996) | **0.019** |  |  |
| Height | 0.985 (0.974 – 0.997) | **0.014** |  |  |
| BMI | 0.94 (0.838 – 1.055) | 0.292 |  |  |
| BMI percentile | 1.023 (1.008 – 1.038 | **0.002** |  |  |
| Undernutrition | 8.667 (2.076 – 36.178) | **0.003** |  |  |
| IBD | 1.117 (0.49 – 2.549) | 0.792 |  |  |
| Crohn's disease | 0.643 (0.136 – 3.035) | 0.577 |  |  |
| Psychological comorbidity | 35.876 (14.33 – 89.815) | **<0.001** | 10.985 (3.069 – 35.763) | **<0.001** |
